# A Virtual Reality Full Body Illusion Improves Body Image Disturbance in Anorexia Nervosa

**DOI:** 10.1371/journal.pone.0163921

**Published:** 2016-10-06

**Authors:** Anouk Keizer, Annemarie van Elburg, Rossa Helms, H. Chris Dijkerman

**Affiliations:** 1 Experimental Psychology/Helmholtz Institute, Utrecht University, Utrecht, The Netherlands; 2 Altrecht Center for Eating Disorders Rintveld, Altrecht Mental Health Institute, Zeist, The Netherlands; 3 Clinical Psychology, Utrecht University, Utrecht, The Netherlands; 4 Department of Neurology, University Medical Centre Utrecht, Utrecht, The Netherlands; Charité-Universitätsmedizin Berlin, Campus Benjamin Franklin, GERMANY

## Abstract

**Background:**

Patients with anorexia nervosa (AN) have a persistent distorted experience of the size of their body. Previously we found that the Rubber Hand Illusion improves hand size estimation in this group. Here we investigated whether a Full Body Illusion (FBI) affects body size estimation of body parts more emotionally salient than the hand. In the FBI, analogue to the RHI, participants experience ownership over an entire virtual body in VR after synchronous visuo-tactile stimulation of the actual and virtual body.

**Methods and Results:**

We asked participants to estimate their body size (shoulders, abdomen, hips) before the FBI was induced, directly after induction and at ~2 hour 45 minutes follow-up. The results showed that AN patients (N = 30) decrease the overestimation of their shoulders, abdomen and hips directly after the FBI was induced. This effect was strongest for estimates of circumference, and also observed in the asynchronous control condition of the illusion. Moreover, at follow-up, the improvements in body size estimation could still be observed in the AN group. Notably, the HC group (N = 29) also showed changes in body size estimation after the FBI, but the effect showed a different pattern than that of the AN group.

**Conclusion:**

The results lead us to conclude that the disturbed experience of body size in AN is flexible and can be changed, even for highly emotional body parts. As such this study offers novel starting points from which new interventions for body image disturbance in AN can be developed.

## Introduction

A key symptom of anorexia nervosa (AN) is a disturbed body representation: Even though patients are underweight, they experience their body as being too fat [1–5]. The disturbed experience of body size in AN is central to the disorder and has been linked to development, prognosis, and maintenance of AN, as well as relapse [6–9]. It is very difficult to treat, and often persists after otherwise successful treatment [10, 11]. This may be related to treatment of body image disturbance in AN focusing mostly on changing bodily cognitions and visual perception of the body, instead of targeting the full multisensory spectrum of the disturbance. We believe that in order to design more effective interventions it is important to understand the disturbed experience of body size from a multisensory perspective. In the current paper we will show that multisensory bodily illusions can play a role in altering misperception of body size in AN.

In the literature, several authors suggest that the representation of our body, which is crucial for how we experience our body and its size, can be divided into separate sub-representations (see e.g. [12–15]). A popular and straight forward distinction is that between body image (a perceptual body representation) and body schema (used for motor action) [16–18]. However, different authors provide different definitions and models of body representation and its (sometimes numerous) sub-representations that may have a hierarchical or more continuous structure (e.g. [12–14, 16]). It is beyond the scope of this paper to review different models of body representation. What *is* relevant however, is that all these models propose that the way in which we experience our body depends on input from multiple sensory modalities. In recent years several authors have shown that understanding the disturbed experience of body size that is typical in AN can be facilitated by adopting a model of body representation in which not only the visual sense is represented, but where there is also room for other senses (for a review see [19]). It has for example been found that body image disturbances in AN also manifest themselves in overestimation of the size of tactile stimuli [20, 21], disturbed haptic perception [22, 23], altered integration of visual and proprioceptive information [24–26], abnormal body scaled action [27, 28], and decreased interoceptive awareness/sensitivity [29, 30]. Moreover, crossmodal integration of sensory signals has been found to be disturbed as well.

Case and colleagues [26] for example showed that AN patients have a diminished Size Weight Illusion (SWI), implying distorted haptic-visual-proprioceptive integration. In their review, Gaudio and colleagues [19] suggest that “AN patients seem to disintegrate physical from subjective dimensions of bodily experience,” but that at this point the underlying processes causing disturbed body size experience in AN are unclear. In other words, we are now at the point at which we *know* that several sensory modalities are affected with regard to body (size) perception and experience in AN, we do however not yet know *why* AN patients process sensory information pertaining the body differently than healthy individuals. Following Gaudio et al’s [19] suggestion, we should now attempt to disentangle the role of different sensory modalities in body size experience, as well as aim to understand the interaction between different sensory modalities.

A first step in understanding multisensory integration in AN was not only made with the SWI [26], but also by using other multisensory bodily illusions such as the Rubber Hand Illusion (RHI). During the RHI the experience of ownership over a fake body (part) is induced. Embodying a fake body (part) results from a visuo-tactile conflict, such as simultaneously touching the actual body (part) that is hidden from view and the fake body (part). Seeing touch on a fake body (part) while at the same time feeling this touch on your actual body causes the brain to integrate these two separate streams of input into one single event, making it seem as if you can feel touch on the fake body (part) [31–33]. Studies in which healthy individuals experienced a rubber hand as belonging to their own body indicate that the location and size of the rubber hand are incorporated in the mental representation of the body [34–36]. The rubber hand is thus replacing the own limb [37].

Interestingly, it was recently found that both acute and recovered AN patients are more susceptible to experiencing the RHI than healthy control participants [24, 25, 38]. More importantly, we found that after inducing the RHI, AN patients’ initial overestimation of their hand width disappeared [25]. Thus although it is difficult to treat body image disturbance in clinical practice, our study shows that it *is* possible to change, normalize even, AN patients’ disturbed experience of body size in an experimental setting using a multisensory body illusion. What is striking about these results is that they do not seem to depend on embodying a fake body part, as improved body size estimation was found in the synchronous condition of the RHI, but also in the asynchronous control condition, in which typically no or less ownership over the rubber hand is experienced [25]. A possible explanation for these findings is that participants based their hand size estimates on the most recent available visual input, i.e. visual input of the rubber hand. In our study we did not include a follow-up measure that took place after the experiment ended and participants had again seen their actual hand. Thus, from our previous RHI study [25], the underlying mechanisms for changing body size estimation in AN using multisensory illusions are not entirely clear. However, in order for these to translate to clinical practice it is foremost important to investigate whether improvements in body size estimation also occur for body parts that are more emotionally salient (i.e. more prone to worry of fatness and dissatisfaction) than the hand, such as the abdomen or the hips.

Therefore, in the current study we induced a bodily illusion in which participants embodied an entire fake, virtual, body. With the emergence of Virtual Reality (VR) techniques, Full Body Illusions (FBI) and body swapping experiments have become an increasingly popular method for investigating how illusory ownership over an entire fake or virtual body affects various aspects of bodily perception and experience [33, 39–48]. Even though healthy individuals have a relatively stable internal representation of their own body [13], in an experimental FBI setting their perception of body size is temporarily malleable (e.g. [40, 45]). Depending on the avatar, individuals might for example experience their body size to be increased [40, 44, 45, 47] or resemble that of a child [39]. Do note that the malleability of body size perception appears limited, at least in healthy populations (see e.g. [45]). However, what happens when ownership over a virtual body is induced in a group of patients who have a distorted experience of body size to begin with?

In the current study participants (AN and healthy females) experienced illusory ownership over a virtual female body with a healthy BMI (see also [49]). Before and after the FBI was induced participants estimated the width and circumference of several parts of their body (height, shoulders, abdomen, hips). Also, a subset of the participants estimated their body size during a follow-up measure, taking place about 2 hours and 45 minutes after the experiment ended. We instructed participants to estimate their body size according to how they subjectively experience/”feel” their body. From clinical experience we know that AN patients often struggle with a discrepancy between knowing their size and feeling their size. Patients often cognitively know that they cannot be fat, e.g. given their weight, but nevertheless they infer from their bodily experiences that they have fat on their body, take up too much space, etc. We wanted to be sure that patients made a size estimation based on their subjective experience of their body size instead of based on conceptual knowledge they might have. In addition Piryankova and colleagues [45] previously showed that body size estimates are sensitive to the specific instructions given. In a healthy sample they found that a FBI affects size estimates of the experienced body (i.e. how participants subjectively “feel” their size), but not estimates of the physical body (i.e. knowledge about body size stored in memory) [45]. These findings might imply that a FBI can update the online representation of body size based on multisensory information (i.e. visuo-tactile integration resulting in ownership), but that more structural knowledge about the body (as stored in memory) is more stable and less susceptible to influences of bodily illusions.

Healthy females were included as a control group, to assess baseline performance. Based on our previous RHI study [25] we did not expect any changes in their size estimation after induction of the FBI. For AN patients however, we expected, again following our previous RHI results [25], that they would overestimate their body size compared to HC before induction of the FBI. Moreover, we expected a decrease in overestimation in AN patients after induction of the FBI. Congruent to our findings with the RHI [25] we expected changes in body size estimation in the AN group to occur after both the synchronous and asynchronous condition. Furthermore, we expected that changes in size estimation after the FBI would occur for all body parts, most strongly for the body parts that are less emotionally salient (shoulders vs abdomen and hips, see also [50]), as previous work indicated that AN patients show distortions in size estimations especially for body parts that are highly emotionally salient [20]. This has been found to be related to negative attitudes about those body parts [20], which may in a top-down fashion affect size estimates, and may still exert an influence after a bodily illusion has been induced. We included the follow-up condition for explorative reasons, as a first step in exploring whether any effects of the FBI on body size estimation in either group would remain over time.

## Methods

### Participants

The current study was approved by the local medical ethical committees of the involved institutions (Medisch Ethische Toetsingscommissie Universitair Medisch Centrum Utrecht protocol id 14-347/D NL49656.041.14 and Commissie Wetenschappelijk Onderzoek Altrecht protocol id 1423). All participants (30 AN and 29 healthy controls (HC)) were female, over 18 years of age, and had no physical conditions that prevented them from performing the tasks. AN patients were recruited at an eating disorder center (Altrecht Eetstoornissen Rintveld, Zeist, The Netherlands). The HC group consisted of students who were recruited on campus (Utrecht University, Utrecht, The Netherlands).

Presence of an eating disorder was excluded in HC by administering the EDI-II. AN patients were diagnosed with the Eating Disorder Examination (EDE) [51] and a psychiatric interview following DSM IV criteria. Patients received treatment as usual which ranged in frequency from daily to weekly sessions. As treatment was aimed at gaining weight, for some patients their initial AN diagnosis changed to EDNOS (N = 6), as they no longer fulfilled the weight and/or amenorrhea criterion for AN at the time of the study.

### Methods, materials & procedure

Before the experiment started participants received verbal explanation of the procedures and provided written informed consent. At the start of the experiment they filled out several questionnaires (demographics, Body Attitude Test (BAT) [52], Eating Disorder Inventory-II (EDI-II) [53]). Afterwards participants estimated the width and circumference of several parts of their body (pre size estimation). Width of the shoulders, abdomen and hips was estimated by placing two adhesive markers on the wall representing the left and right side of the body. Circumference was estimated using a piece of string that was placed on the floor so that it would fit exactly around each body part. Participants were explicitly instructed to estimate the width/circumference of their own body (e.g. “Please place two adhesive markers on the wall so that your hips exactly fit in between the markers, your size estimation should represent how you experience your body size”). In addition, participants estimated their height using an adhesive marker they placed on the wall. The order of body parts (height, shoulders, abdomen, hips) and type of estimation (width, circumference) was counterbalanced over participants.

**Fig 1:**
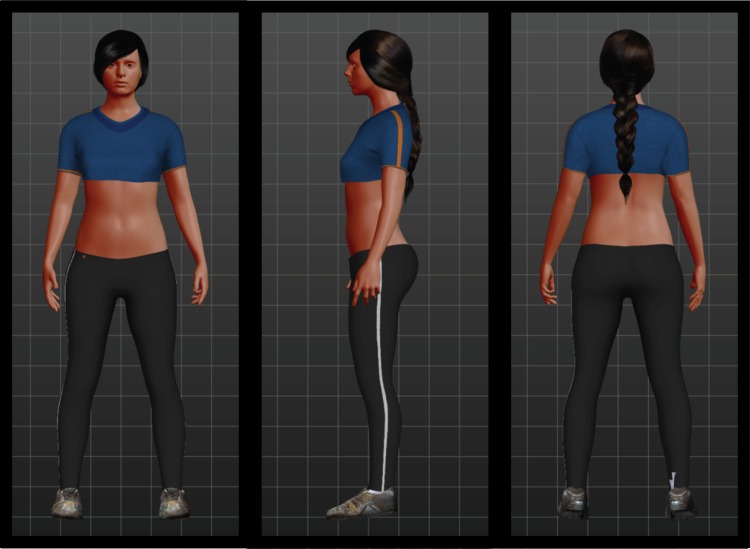
Front, side and back view of the avatar from a third person perspective.

Subsequently the FBI was induced twice, once with synchronous visuo-tactile stimulation (experimental condition) and once with asynchronous visuo-tactile stimulation (control condition) (see also [49]), the order of conditions was counterbalanced over participants. We used Unity3D (unity3d.com) to set up the VR scene. The room was built directly in Unity using textured cubes. The hand model holding a brush was provided by Sixense in their Unity plugin (https://www.assetstore.unity3d.com/en/#!/content/7953). We edited the hand's texture to have less pronounced veins. The brush was modelled in Blender (https://www.blender.org/) to look as much as the real brush as possible. The avatar was created using MakeHuman (http://www.makehuman.org/), see Fig 1 for a third person perspective of the avatar. MakeHuman uses several parameters that influence the body. We set the age of the avatar to 25 and all parameters to default/neutral which resulted in a waist to hip ratio of 0.75, and a waist circumference of 71.83cm, which is considered healthy by the World Health Organisation [54].

**Fig 2:**
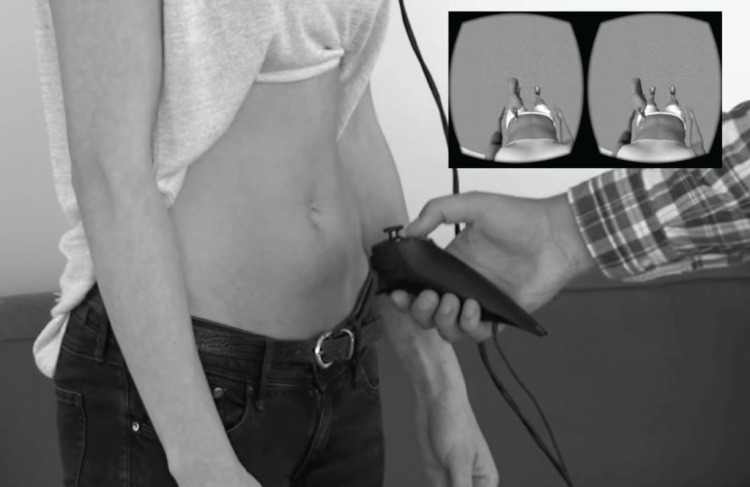
Experimental set up. Experimenter touching participants’ actual abdomen with a movement sensor, stroking movements are displayed in VR (inlay).

To induce the illusion participants were asked to bare their abdomen and put on the VR goggles (Oculus Rift DK2). They were then instructed to look down, till they saw (from a first person perspective) the body of the avatar (see Fig 2). The experimenter stroked the actual abdomen of the participant from the stomach towards the belly button in downward movements for 90 sec with a soft brush. The brush was attached to a movement sensor (Razer Hydra), which communicated with the VR software, so that the participants saw the stroking movements made by the experimenter on her actual abdomen mimicked on the abdomen of the avatar (see Fig 2). The latency from brush movement to visible change was about 100 milliseconds. Such a delay is noticeable during fast movement. However, during the experiment fast movements were avoided and there was no noticeable mismatch in observed and felt position due to latency. Depending on the condition, stroking on the actual abdomen and virtual abdomen took place simultaneous (synchronous condition) or was delayed in VR (asynchronous condition). The asynchronous condition was similar to asynchronous stroking in the RHI, in which stroking of the participants’ hand is alternated with stroking the rubber hand. Here stroking of the participants’ abdomen was recorded by pressing a button on the motion sensor. This froze the image seen by participants in VR (i.e. participants felt the brush stroking their abdomen, but did not see it taking place on the abdomen of the avatar in VR), the stroking movement was replayed in VR as soon as the experimenter finished stroking and released the button on the movement sensor (i.e. now participants did not feel the brush touching their abdomen, but did see it touching the abdomen of the avatar).

**Fig 3:**
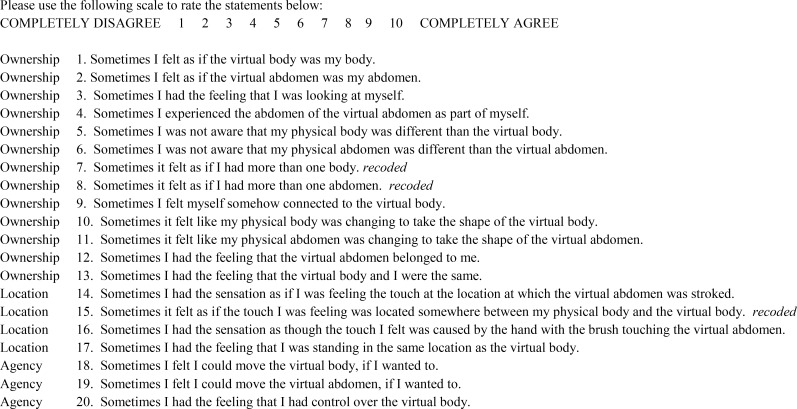
Embodiment Questionnaire (EQ, based on [45]) assessing the strength of the illusion on three subscales.

After taking off the VR goggles participants again estimated the size of their body (post size estimation), in the same way as they made body size estimations before the FBI was induced. Furthermore, they filled in the Embodiment Questionnaire (EQ), assessing how they subjectively experienced the illusion (see Fig 3, based on [45]). The EQ consisted of 20 items that made up three subscales measuring 1) ownership over the virtual body; 2) being in the same location as the virtual body; and 3) experiencing agency over the virtual body. Participants rated each statement on a 10-point Likert scale, with higher scores indicating a stronger illusion. After the procedure for the FBI was completed twice (synchronous and asynchronous condition), the experimenter measured participants’ actual body dimensions and weight. A subset of the participants estimated their body size again during a follow-up measure, taking place about 2 hours and 45 minutes after the experiment ended (N_AN_ = 9, time M_AN_ = 155.77mins, SD = 43.08; N_HC_ = 26, time M_HC_ = 191.04mins, SD = 97.56, t(30.39) = -1.47, p = .153).

### Statistics

A power analysis was conducted with G*power 3.1.9.2, in which we calculated the a-priori sample size for comparing the means of two groups with a mixed repeated measures ANOVA. We based the effect size *f* on our previous findings of changes in hand size estimation in AN patients after a bodily illusion (i.e. the RHI) [25]. In order to obtain a power of 0.80, with alpha set at .05, and *f* at 0.33, a total sample size of 58 participants, was required, 29 in each group.

We assessed differences between AN patients and HC in demographical data and eating disorder pathology assessment using Bonferroni corrected independent samples t-tests, and, as data for assessment of highest weight never was not normally distributed, a Mann-Whitney U test.

The subjective experience of the FBI was assessed with the EQ. Differences within and between groups were tested using a 3 (Subscale; ownership vs location vs agency) x 2 (Condition; synchronous vs asynchronous) x 2 (Group; AN vs HC) repeated measures ANOVA. When relevant, significant interactions were broken down using Bonferroni corrected post-hoc tests.

The difference in actual body size between AN patients and HC was tested with Bonferroni corrected independent samples t-tests and, as data for height was nog normally distributed, a Mann Whitney U test.

Participants estimated their body size before and after the FBI, and at follow-up. We calculated the percentage of misestimation (i.e. either over- or underestimation) for each body part of interest using the following formula: percentage of misestimation = ((estimated size–actual size)/actual size)*100. With 3 (Condition; pre vs post synchronous vs post asynchronous) x 2 (Group; AN vs HC) repeated measures ANOVAs we compared AN and HC’s percentage of misestimation at pre and post FBI for each body part separately (height, shoulder width; abdomen width; hip width; shoulder circumference; abdomen circumference; and hip circumference). When relevant, significant interactions were broken down using Bonferroni corrected post-hoc tests.

Follow-up data was not collected in the full sample, therefore those data were analyzed separate from the pre and post illusion data. After calculating the percentage of misestimation at follow-up, we conducted Bonferroni corrected Mann Whitney U tests to compare the AN and HC group. Changes from pre to follow-up illusion within each group were tested using Bonferroni corrected Wilcoxon Signed Rank tests. We opted for nonparametric testing as group size was small and unequal for follow-up data.

## Results

### Demographics and eating disorder pathology

Independent samples t-tests showed that AN patients and HC did not differ from each other in age. AN patients had a lower BMI than HC, as well as a lower lowest and highest weight ever. AN patients worried more about their abdomen and hips being too fat, and their shoulders being too wide than HC, they were also more dissatisfied with their height than HC (see Table 1, also for additional clinical characteristics of the patient group).

**Table 1:** Means and SD’s of demographic and clinical variables by group, and results of comparing groups on these variables.

	AN patients (n = 30)		HC (n = 29)					
	M	SD	M	SD	t	df	p	d
Age (years)	22.03	3.67	21.07	2.34	1.21	49.50	.233	0.31
BMI	18.11	1.68	20.77	1.48	-6.45	57	< .001	1.68
Lowest weight ever	42.20	5.82	57.88	7.46	-9.02	57	< .001	1.20
Highest weight ever _Mann-Withney U test_	61.73	8.23	66.16	6.84	245.50	-2.74	.006	0.33
Fat %	16.04	7.04	n/a	n/a	n/a	n/a	n/a	n/a
Weight at intake	45.74	5.47	n/a	n/a	n/a	n/a	n/a	n/a
BMI at intake	16.29	1.76	n/a	n/a	n/a	n/a	n/a	n/a
Fat % at intake	8.81	6.43	n/a	n/a	n/a	n/a	n/a	n/a
Illness duration (months)	10.61	11.62	n/a	n/a	n/a	n/a	n/a	n/a
Worry abdomen too fat	8.30	1.78	4.83	1.97	7.10	56.04	< .001	2.18
Worry shoulders too wide	5.53	2.80	2.59	1.66	4.94	47.40	< .001	1.28
Worry hips too fat	8.47	1.57	3.76	2.18	9.54	57	< .001	2.48
Dissatisfaction with height	5.00	2.63	2.93	1.93	3.46	53.19	.001	0.90
Total BAT score	82.40	16.65	39.76	8.05	12.59	42.16	< .001	2.46
BAT negative appreciation body	34.77	7.58	15.66	3.88	12.25	43.50	< .001	3.17
BAT lack familiarity with body	23.17	7.89	8.62	3.05	9.61	38.18	< .001	2.49
BAT general dissatisfaction body	2.12	0.72	3.90	0.47	12.57	57	< .001	2.79
Total EDI-II score	243.83	37.13	156.38	32.78	9.58	57	< .001	2.50
EDI drive for thinness	34.47	7.14	17.52	5.36	10.29	57	< .001	1.42
EDI bulimia	11.63	3.76	14.10	4.64	-2.25	57	.028	0.58
EDI body dissatisfaction	45.07	7.65	24.17	6.75	11.11	57	< .001	2.90
EDI ineffectiveness	40.93	9.11	22.52	6.44	8.94	57	< .001	2.33
EDI perfectionism	23.93	6.01	16.62	3.97	5.50	57	< .001	0.68
EDI interpersonal distrust	23.87	4.85	17.07	4.47	5.59	57	< .001	1.46
EDI interoceptive awareness	36.40	8.15	22.24	6.93	7.18	57	< .001	1.87
EDI maturity fears	27.53	6.54	22.14	5.56	3.41	57	.001	0.89

Note. The variable “highest weight ever” was not normally distributed, group differences were tested using a non-parametric Mann-Whitney U test; Mann-Whitney U value is reported in the “t” column, Z score is reported in the “df” column.

We assessed body dissatisfaction with the (BAT), results of independent samples t-tests indicated that AN patients showed a higher total score as well as higher scores on all subscales of the BAT compared to HC. Similar results were found for the EDI-II assessing eating disorder pathology. Independent samples t-tests showed that AN patients had a higher total score and higher scores on all subscales than HC, except for the Bulimia subscale (see Table 1).

### Subjective experience of the full body illusion

We assessed the subjective experience of the FBI with the EQ (based on [45]; see Fig 2), consisting of an ownership, location and agency subscale. A repeated measures ANOVA showed a main effect for Subscale (ownership vs location vs agency), F(2, 56) = 9.15, p < .001, ɳ^2^ = 0.25; a main effect for Condition (synchronous vs asynchronous); F(1, 57) = 39.44, p < .001, ɳ^2^ = 0.41, and an interaction between Subscale and Condition (F(2, 56) = 7.59, p = .001, ɳ^2^ = 0.21. No main effect for Group (AN vs HC) was found, F(1, 57) = 0.08, p = .773, ɳ^2^ = 0.00. Post-hoc paired samples t-tests showed that, independent of group, participants gave higher ratings on the EQ for the ownership subscale in the synchronous compared to the asynchronous condition t(58) = 1.17, p < .001, d = 0.60. Similar results were found for the location subscale, t(58) = 1.88, p < .001, d = 1.02, and the agency subscale, t(58) = 1.90, p = .004, d = 0.40.

Taken together these results show that AN patients and HC had an equally strong experience of the FBI, and as assumed both groups experienced more ownership and agency over the virtual body and a stronger shift in location towards the virtual body in the synchronous compared to the asynchronous (control) condition. Means and SD’s by subscale, condition, and group can be found in Table 2.

**Table 2:** Means and SDs of Embodiment Questionnaire (EQ) subscales scores by group for the synchronous (experimental) and asynchronous (control) condition.

	AN patients (n = 30)		HC (n = 29)	
	*M*	*SD*	*M*	*SD*
Ownership sync	5.40	1.90	5.03	2.10
Location sync	6.13	1.73	6.36	1.31
Agency sync	4.79	2.86	5.22	2.43
Ownership async	4.01	1.93	4.08	1.96
Location async	4.39	2.07	4.61	1.71
Agency async	4.18	2.76	4.34	2.41

### Actual body size of participants

Independent samples t-tests and a Mann-Whitney U test showed that AN had smaller body height, width, and circumference dimensions than HC, except for shoulder width (see Table 3).

**Table 3:** Means and SD’s for actual body size by group, and comparisons between AN and HC group.

	AN patients (n = 30)		HC (n = 29)					
	M	SD	M	SD	t	df	p	d
Height* _Mann-Withney U test_	167.68	4.79	171.71	6.24	232.00	-3.09	.002	0.72
Shoulder width	39.37	1.77	40.27	1.66	-2.02	57	.048	0.52
Abdomen width	26.22	2.03	28.20	2.39	-3.44	57	.001	0.89
Hip width	32.53	2.12	35.15	2.76	-4.09	57	< .001	1.06
Shoulder circumference	95.93	4.11	100.03	4.66	-3.59	57	.001	0.93
Waist circumference	70.50	5.02	75.97	6.15	-3.74	57	< .001	0.97
Hip circumference	88.73	5.67	97.93	6.44	-5.83	57	< .001	1.52

Note. The variable “height” was not normally distributed, group differences were tested using a non-parametric Mann-Whitney U test; Mann-Whitney U value is reported in the “t” column, Z score is reported in the “df” column.

### Pre vs post FBI body width estimation

#### Height

A repeated measures ANOVA for height estimation showed no main effect for Condition, nor Group. The interaction between Condition and Group was not significant (see Table 4).

**Table 4:** Results of repeated measures ANOVA’s for within and between group differences in percentage of misestimation of body size.

		F	df	*p*	ɳ^2^
Height					
	Main effect Condition (pre vs synchronous vs asynchronous)	1.89	2,56	.160	0.06
	Main effect Group (AN vs HC)	0.05	1,57	.830	0.00
	Condition*Group	1.85	2,56	.166	0.06
Shoulder width					
	Main effect Condition (pre vs synchronous vs asynchronous)	14.16	2,56	< .001	0.34
	Main effect Group (AN vs HC)	23.50	1,57	< .001	0.29
	Condition*Group	1.24	2,56	.298	0.04
Abdomen width					
	Main effect Condition (pre vs synchronous vs asynchronous)	0.15	2,56	.860	0.01
	Main effect Group (AN vs HC)	25.61	1,57	< .001	0.31
	Condition*Group	0.00	2,56	.999	0.00
Hip width					
	Main effect Condition (pre vs synchronous vs asynchronous)	4.10	2,56	.022	0.13
	Main effect Group (AN vs HC)	24.56	1,57	< .001	0.30
	Condition*Group	0.58	2,56	.565	0.02
Shoulder circumference					
	Main effect Condition (pre vs synchronous vs asynchronous)	23.83	2,56	< .001	0.46
	Main effect Group (AN vs HC)	33.57	1,57	< .001	0.37
	Condition*Group	0.92	2,56	.404	0.03
Abdomen circumference					
	Main effect Condition (pre vs synchronous vs asynchronous)	4.23	2,56	0.19	0.13
	Main effect Group (AN vs HC)	42.27	1,57	< .001	0.43
	Condition*Group	3.03	2,56	.056	0.10
Hip circumference					
	Main effect Condition (pre vs synchronous vs asynchronous)	24.26	2,56	< .001	0.46
	Main effect Group (AN vs HC)	42.14	1,57	< .001	0.43
	Condition*Group	0.83	2,56	.439	0.03

#### Shoulder width

A repeated measures ANOVA for shoulder width estimation showed a main effect for Condition and for Group. The interaction between Condition and Group was not significant. Post hoc pairwise comparisons for the main effect of Condition show that, independent of group, participants’ misestimation of shoulder width significantly decreases from pre to post synchronous size estimation (p < .001) as well as from pre to post asynchronous size estimation (p < .001), while there was no difference in misestimation between post synchronous and post asynchronous size estimation (p = 1.000). In addition, independent of condition, AN patients show larger percentages of misestimation of shoulder width than HC.

#### Abdomen width

A repeated measures ANOVA for abdomen width estimation showed no main effect for Condition, but the main effect for group was significant Group. The interaction between Condition and Group was not significant. Thus, independent of condition, AN patients showed larger percentages of misestimation of abdomen width than HC.

#### Hip width

A repeated measures ANOVA for hip width estimation showed a main effect for Condition and Group. The interaction between Condition and Group was not significant. Post hoc pairwise comparisons for the main effect of Condition show that, independent of group, participants’ misestimation significantly decreases from pre to post synchronous size estimation (p = .026), while there was no difference from pre to post asynchronous size estimation (p < .155), nor between post synchronous and post asynchronous size estimation (*p* = 1.000). Further, independent of condition, AN patients show larger percentages of misestimation than HC.

### Pre vs post FBI body circumference estimation

#### Shoulder circumference

A repeated measures ANOVA for shoulder circumference estimation showed a main effect for Condition and Group. The interaction between Condition and Group was not significant. Post hoc pairwise comparisons for the main effect of Condition show that, independent of group, participants’ misestimation of shoulder circumference significantly decreases from pre to post synchronous size estimation (p < .001), as well as from pre to post asynchronous size estimation (p < .001), while there was no difference in misestimation between post synchronous and post asynchronous size estimation (p = 1.000). In addition, independent of condition, AN patients show larger percentages of misestimation of shoulder circumference than HC.

#### Abdomen circumference

A repeated measures ANOVA for abdomen width estimation showed a main effect for Condition and Group. The interaction between Condition and Group was marginally significant. Independent of condition, AN patients showed larger percentages of overestimation than HC. When breaking down the interaction between Condition and Group, paired samples t-tests showed that for AN patients the percentage of misestimation of abdomen width significantly decreased from pre to post synchronous illusion, t(29) = 3.03, p = .005, d = 0.41, and marginally significantly decreased from pre to post asynchronous illusion, t(29) = 2.35, p = .026, d = 0.28. There was no difference in misestimation between post synchronous illusion and post asynchronous illusion, t(29) = -1.24, p = .223, d = 0.16. For HC there was no significant difference from pre to post synchronous illusion, t(29) = 0.35, p = .730, d = 0.05, from pre to post asynchronous illusion, t(29) = 0.47, p = .639, d = 0.08, nor between post synchronous illusion and post asynchronous illusion, t(29) = 0.15, p = .883, d = 0.02.

#### Hip circumference

A repeated measures ANOVA for hip circumference estimation showed a main effect for Condition and Group. The interaction between Condition and Group was not significant. Post hoc pairwise comparisons for the main effect of Condition show that, independent of group, participants’ misestimation of hip circumference significantly decreases from pre to post synchronous size estimation (p < .001), as well as from pre to post asynchronous size estimation (p < .001), while there was no difference is misestimation between post synchronous and post asynchronous size estimation (p = 1.000). Further, independent of condition, AN patients show larger percentages of misestimation of hip circumference than HC.

Taken together the results show that only for abdomen circumference estimates AN patients showed a larger decrease in percentage of misestimation from pre to post synchronous as well as a marginally significant decrease from to post asynchronous illusion than HC (see Table 5 for means and SD’s by group at pre, post synchronous and post asynchronous illusion). For all the other body parts, except height and abdomen width, both AN patients and HC showed a significant decrease in percentage of misestimation from pre to post illusion, while size estimates in the two post illusion conditions (synchronous vs asynchronous) did not differ from each other. In addition, for all body parts, except height, AN patients showed larger percentages of misestimation compared to HC.

**Table 5:** Means and SD’s of pre-illusion, post-illusion (synchronous and asynchronous condition), and follow-up illusion percentage of misestimation of body size by group.

		AN (n = 30)		HC (n = 29)	
Pre FBI % misestimation		M	SD	M	SD
	height	0.97	2.98	1.57	2.67
	shoulder width	16.92	19.68	-3.70	12.42
	abdomen width	43.26	43.89	6.06	14.75
	hip width	35.01	31.79	1.73	10.74
	shoulder circumference	28.58	12.92	8.01	9.87
	abdomen circumference	60.46	25.37	21.02	11.97
	hip circumference	40.33	21.11	10.15	9.92
Post FBI sync % misestimation		M	SD	M	SD
	height	0.94	2.79	0.53	2.13
	shoulder width	7.65	18.61	-11.80	13.04
	abdomen width	42.63	36.98	5.71	18.68
	hip width	29.81	34.37	-0.97	13.05
	shoulder circumference	19.00	15.54	1.67	11.06
	abdomen circumference	48.94	30.42	20.26	17.17
	hip circumference	30.25	21.95	2.80	12.71
Post FBI async % misestimation		M	SD	M	SD
	height	0.90	2.29	1.07	2.23
	shoulder width	6.68	18.62	-9.16	13.88
	abdomen width	41.79	37.16	4.92	17.16
	hip width	29.40	34.20	-0.25	13.54
	shoulder circumference	18.66	16.88	0.65	13.60
	abdomen circumference	53.50	25.15	19.97	15.38
	hip circumference	30.53	22.31	3.70	11.89
Follow-up FBI % misestimation		M	SD	M	SD
	height	1.59	3.12	-0.21	2.48
	shoulder width	-2.39	11.59	-7.71	12.02
	abdomen width	38.86	19.19	3.19	17.29
	hip width	26.37	11.03	0.32	12.99
	shoulder circumference	11.94	12.25	4.13	13.54
	abdomen circumference	40.33	19.92	14.72	14.72
	hip circumference	19.94	16.68	4.88	10.39

### Follow-up body size estimation

About 2 hours and 45 minutes after the FBI a subset of the participants estimated height, width, and circumference of their body again (N_AN_ = 9; N_HC_ = 26).

Mann Whitney U tests showed that at follow-up AN patients made larger estimation errors than HC for abdomen width, hip width, abdomen circumference and (marginally significant for) hip circumference (all *p*’s < .050). No differences were found at follow-up between the two groups for percentage of misestimation of height, shoulder width, and shoulder circumference (all p’s >.152; see Table 5).

Wilcoxon signed ranks tests showed that from pre to follow-up illusion AN patients’ percentage of misestimation decreased for shoulder width, z = -2.67, p = .008, d = 1.20, but not for height, z = -0.18, p = .859, d = .020, not for abdomen width, z = -1.36, p = .173, d = 0.13, and not for hip width, z = -1.84, p = .066, d = .036. From pre to follow-up HC showed a marginally significant decrease in percentage of misestimation for height, z = -2.19, p = .029, d = 0.69, but not for shoulder width, z = -1.21, p = .228, d = 0.33, not for abdomen width, z = -0.92, p = .360, d = 0.18, and not for hip width, z = -1.05, p = .294, d = 0.12.

Wilcoxon signed ranks tests showed that from pre to follow-up illusion AN patients’ percentage of misestimation decreased for shoulder circumference, z = -2.31, p = .021, d = 1.32 (marginally significant), and hip circumference, z = -2.55, p = .011, d = 1.07, but not for abdomen circumference, z = -1.54, p = .123, d = 0.89. HC showed a decrease in percentage of misestimation of abdomen circumference, z = -2.13, p = .033, d = 0.47 (marginally significant), and hip circumference, z = -2.93, p = .003, d = 0.52, but not for shoulder circumference, z = -1.58, p = .114, d = 0.33.

Taken together these findings show that approximately 2 hours and 45 minutes after the illusion was induced participants still showed changed perception of body size, especially the AN group, and for estimates of circumference of the body. AN patients showed a decrease in percentage of misestimation of shoulder width, shoulder circumference, and hip circumference. HC showed a decrease in percentage of misestimation of height, abdomen circumference, and hip circumference. In the AN group changes in percentage of misestimation at follow-up were found for 3 out of the 5 body parts for which an effect was also found immediately after the FBI. For HC changed body size estimation followed a different pattern, as an effect for height and abdomen circumference was found only at follow-up, not immediately after the FBI, while a decrease in percentage of misestimation for hip circumference was found both post illusion and at follow-up.

## Discussion

We previously found that after inducing the RHI patients with AN estimated their hand size more accurately compared to before the illusion [25]. Our main question in the current study was whether this effect would also occur for body parts more emotionally salient than the hand after inducing a FBI in which participants experienced ownership over a virtual body (see also [49]). Following our previous RHI study [25] we expected AN patients to overestimate body size before the FBI, and a decrease in the amount of misestimation of body size after the illusion was induced.

### Baseline body size estimation

We expected that before the FBI was induced AN patients would overestimate their body size compared to HC. Our results confirmed this hypothesis: AN patients showed larger percentages of misestimation for their body width and body circumference, but not for their height. This indicates that overestimation of body size in AN is not the result of a general bias in estimating the own body as bigger than it actually is, but is specifically limited to experiencing the own body as wider and rounder, i.e. fatter. Effect sizes indicate that the differences in size estimation errors between the AN and HC group are more profound for body circumference than body width.

### Body size estimation after the FBI and at follow-up

Following previous RHI findings [25] we hypothesized that AN patients would show a decrease in percentage of misestimation of body size after induction of the FBI in the synchronous and asynchronous condition, especially for less emotionally salient body parts, while we expected HC to remain stable in their size estimates. This hypothesis was partly confirmed by the results. After the FBI was induced we found a *decrease* in the percentage of misestimation from pre to post illusion in the synchronous and asynchronous condition in AN group, but also in the HC group. For most body parts of interest the AN and HC group showed decreased misestimation of similar magnitude. The only exception was that AN patients showed a decrease in circumference estimates of their abdomen, while in HC this effect was absent. Overall, AN patients did however still show larger percentages of overestimation of body size compared to HC, implying that their body size estimates had not “normalized”.

We wanted to explore whether any changes in body size estimation from pre to post FBI would also remain over time. Therefore a subset of the participants completed a follow-up measure ~2 hours and 45 minutes after the FBI was induced. At follow-up AN patients’ size estimates of their shoulder width and circumference had normalized and no longer significantly differed from HC’s percentages of misestimation. In addition AN patients also showed decreased overestimation of hip circumference. Even though HC also showed a decreased percentage of misestimation of body size at follow up for some body parts (abdomen and hip circumference), the effect sizes indicate that the changes from pre to follow-up illusion were larger in the AN group (all d’s for (marginally) significant effects >1.07) than in the HC group (all d’s for (marginally) significant effects <0.69). Again, AN patients still showed larger percentages of overestimation of body size compared to HC, but only for abdomen and hip estimations, not for shoulder estimates.

Taken together these results show that an experimental FBI setting alters how individuals perceive their body size, independent of whether participants experienced ownership over the virtual body or not. Previously, altered body size experience after a FBI has been reported in healthy subjects (e.g. [45]). Importantly, we observed changed body size experience in AN patients in the current study, whose body size experience is severely disturbed, and very difficult to change in a clinical setting. Nevertheless, the experiment positively affected AN patients’ persistent disturbed experience of body size. After the FBI initial (i.e. before induction of the illusion) overestimation of body size decreased, especially for circumference estimates. Improved body size estimation was observed even at follow-up, ~2 hours and 45 minutes after the illusion was induced. Even though the observed change in body size estimation after the FBI in the HC group was contrary to our hypothesis, the results do confirm our core expectation: It is possible to change AN patients’ disturbed experience of body size for both emotional *and* non-emotional body parts using an FBI setting.

### The role of embodiment in changed body size estimation

What is particularly striking about our findings is that changes in body size estimation not only occurred after the synchronous condition of the FBI, in which participants embodied the avatar, but also in the asynchronous control condition, in which embodiment was significantly lower/absent. According to theoretical models on multimodal bodily experience, embodiment of a fake body (part) does not result in the fake body (part) simply being added to the representation of the body, but in the fake body (part) *replacing* the actual body (part) in the body representation [35–37]. It is suggested that in order for the representation of the body, and the experience of the (size of the) body to change after body illusions, embodiment of a fake body (part) is crucial [34, 36]. Our present FBI findings contradict this reasoning, as changes in body size experience occurred after both the synchronous and asynchronous control condition.

Our findings are in accordance with those found by Piryankova and colleagues [45] in a healthy sample. However, it contradicts two other studies with healthy participants. Normand and colleagues [40] observed a change in body size estimation only after synchronous stimulation, while Preston and colleagues [47] found no change in size estimation after both synchronous and asynchronous stimulation. These inconsistent findings might result from differences in experimental design, such as the method of body size estimation, the level of immersiveness of the FBI, and/or the specific method of inducing the illusion.

Several studies suggest that in an immersive VR environment first person perspective of an avatar with common human features is sufficient for embodying the avatar [45, 55]. Congruent multisensory cues (visuo-tactile or visuo-motor) are not a requirement for the FBI to occur, but they may strengthen the illusion [55]. Nevertheless, our participants subjectively reported more embodiment of the avatar after synchronous stroking, but did not show larger amounts of changes in body size estimation after this stroking condition compared to asynchronous stroking, where they reported less/no embodiment. This seems to imply that embodiment of an avatar in itself is not crucial for changing the experience of body size.

Perhaps the key-element in the current study that caused changed body size estimation in AN patients was removing visual feedback of the participant’s *own* body. Even though participants experienced ownership over the avatar, they knew it was not their own body they were viewing. A study by Øverås and colleagues [56] shows that AN patients overestimate their body size with about 10% more when they directly perceive their body in a mirror, compared to making a size estimate from memory. In our study we observe a decrease in size estimation in the AN group that is similar in magnitude (10%) from pre to post illusion estimates of the abdomen and hips, but only for circumference estimates. The decrease in width estimates was smaller. Øverås and colleagues [56] further showed that healthy participants’ body size estimates were not affected by the available visual input (i.e. estimate from a mirror vs from memory) but remained stable over the two conditions. In contrast, we observed in the current study that in HC body size estimates *did* change and were not stable. This makes it unlikely that only the removal of visual information in our experiment was responsible for changes in body size estimation.

Another possibility is that participants based their size estimates on the most recent visual information that was provided to them, i.e. the body of the avatar. This might also explain why we also found some changes in body size estimation in the HC group. On the other hand this alternative explanation is in conflict with two of our findings. First, participants also showed changes in size estimation of their shoulders, which were not visible in the VR set-up, making it unlikely that participants used visual information of the avatar’s body size for their body size estimate. Interestingly, the shoulders showed the most consistent changes in size estimation after the FBI in the AN group: Misestimation of shoulder width and circumference decreased after the synchronous and asynchronous condition, and at follow-up. Perhaps this consistent change is related to the shoulders being the least emotionally salient body part that was included in the experiment. Second, we observed changes even ~2 hours and 45 minutes after the illusion was induced. In this time frame participants were allowed to leave the lab. Participants will thus have had access to visual feedback on their actual body size before they completed the follow-up measure, making the avatar not the most recent-visual input about body size anymore.

### Future directions

To deepen our insight in the underlying mechanisms of changed body size perception after a FBI, future studies should focus on teasing apart the role of the different sensory modalities involved. Does body size estimation still change after only providing participants with visual information of an avatar, or only with tactile input and no visual information, or is a combination of visual and tactile information required, even if input is not in sync?

Further, in the current study all participants were presented with the same body. For HC the BMI of the avatar was similar to their own BMI (i.e. in the healthy range). The avatar had however bigger body dimensions than the actual body of most AN patients. Strikingly, observations during testing indicate that despite being slimmer than the avatar, many patients spontaneously mentioned “Oh! I am so skinny right now!” when first seeing the avatar while wearing the oculus. It would be interesting to examine the effect of different avatar sizes on subsequent body size estimates. Would the altered body size estimation found here have a different magnitude when the avatar is modelled according to actual body dimensions of the participant? Perhaps more interesting, what would be the effect of letting participants model the avatar themselves, so that it represents their subjectively experienced body size.

What also should be addressed here is that body size estimates improved mainly for estimates of body *circumference* and to a lesser extent for body *width*, i.e. participants estimated their body to be less round. Could it be that width and circumference measure different aspects of size experience? With width reflecting a more abstract, 2D, representation of body size, while circumference taps more into a more coherent, volumetric, 3D representation of the body (see also [13])? And is either one more related to the conscious experience of body size? The current data do not offer a conclusive answer to this matter. A recommendation for future studies is therefore to also assess subjective body attitudes, to gain insight in whether participants emotionally feel different in terms of size and fatness after the illusion (as done by [47]). It is yet unclear whether changes in body size estimation after the FBI run in parallel with changes in emotional/cognitive experience of body size.

### Implications for clinical understanding of body image disturbance in AN

From previous research we know that FBI can temporarily alter an individual’s experience of body shape and size (e.g. [39, 40, 44, 45, 48]). In the introduction we posed the question of what happens with body size estimation after a FBI when body size experience is distorted to begin with, as is the case in AN patients. Our results indicate that the illusion of embodying an avatar basically has no effect. Changes in body size estimation that we found were identified after synchronous stroking, where the avatar was embodied, but also after the asynchronous condition, where there was no/less embodiment. Nevertheless, our findings do show that body image disturbances in AN are malleable in an experimental setting, and that improvements in body size estimation remain over a period of a few hours. Note however, that in healthy participants we also observed a decrease in body size estimation.

As embodiment of the avatar did not appear to be crucial for the changes in body size estimation that we found, we can only speculate why both HC and AN patients estimated their body size differently after the experimental procedures compared to the start of the experiment. Perhaps a certain aspect of the experimental setting was the factor driving changed body size estimation. Participants were put in an experimental setting in which they were forced to use input other than vision of their *own* body to construct a representation of their body size. They were specifically instructed to estimate their body size according to how they subjectively experienced/”felt” their size. Perhaps forcing participants to rely on information from other senses than the visual sense (i.e. tactile, proprioception) when making a size estimate of their body results in a smaller experience of body size.

We further speculate that for AN patients specifically another mechanism might play a role as well, Improved body size estimation after blocking visual input of the own body might be related to how AN patients literally look at themselves. It has been found that AN patients have weaker central coherence compared to healthy females; AN patients show poorer global processing and there are indications of superior local (detail-focused) processing of (visual) information (for reviews see e.g. [57, 58]). Placing this in the light of looking at the own body, it could be that AN patients have a bias towards focusing mostly on specific body parts instead of the global image they see (i.e. the body as a whole). In other words, it could be that such a bias in processing of visual information in AN largely accounts for overestimation of body size typically observed in this group. Once visual information is removed–such as when wearing a VR headset—this negative bias in viewing behaviour is perhaps removed as well, and other sources of input might need to be prioritized over vision when constructing an experience of body size. Possibly processing of information from these other sources of input (e.g. proprioception, interoceptive signals, tactile information) does allow for a more holistic experience of the body, which in turn might improve body size estimation. In relation to this, we know from previous work that processing of bodily input from senses other than vision is disturbed in AN (for a review see [19]), which fits with our current finding that overestimation of body size in AN is decreased, but not fully restored, i.e. patients continue to overestimate their body size compared to HC.

The current study showed that after blocking visual input of the own body, participants estimate their body size as smaller. For clinical practise these findings imply that it may be helpful to let AN patients (consciously) shift their focus from visual information about their body to information from other senses. A training in which patients actually learn to “feel” their body might be useful. However, caution is warranted here, as clinical experience shows that letting AN patients actually “feel” their body, and focus on internal signals from the body, can trigger stress and high levels of anxiety. Such a particular intervention should thus include a slow built-up in “feeling” the body, and also focus on reducing excess feelings of stress or fear. Note that this suggested training does not have to involve a (VR) bodily illusion. We observed changed body size estimation in both the experimental and control condition of the illusion. The illusion itself does not seem to be associated with improved body size estimation, the experimental setting in which it is conducted (i.e. no access to visual information of the own body while at the same time being touched on the body) seems more important.

### Conclusion

Taken together the results imply that it is possible to decrease AN patients’ overestimation of body size in an experimental FBI setting, with effects remaining at least up to ~2 hours and 45 minutes after the illusion was induced. Note that in HC we observed a largely similar decrease in percentage of misestimation of body size. From the results it is yet unclear what the exact underlying mechanism for this change in body size estimation is. This study does however offer important insights in the flexibility of body size experience in AN: It is not static and we *can* change it, even for highly emotional body parts. As such it offers much needed novel directions for understanding body image disturbances in AN. At the same time it provides a starting point for designing new body image disturbance interventions, which is crucial, given the persistent nature of the disturbed experience of body size in AN [10, 11, 38, 59, 60] and its central role in AN pathology [6–9, 61].
